# How has Expenditure on Nicotine Products Changed in a Fast-Evolving Marketplace? A Representative Population Survey in England, 2018–2022

**DOI:** 10.1093/ntr/ntad074

**Published:** 2023-05-25

**Authors:** Sarah E Jackson, Harry Tattan-Birch, Lion Shahab, Jamie Brown

**Affiliations:** Department of Behavioural Science and Health, University College London, London, UK; SPECTRUM Consortium, UK; Department of Behavioural Science and Health, University College London, London, UK; SPECTRUM Consortium, UK; Department of Behavioural Science and Health, University College London, London, UK; SPECTRUM Consortium, UK; Department of Behavioural Science and Health, University College London, London, UK; SPECTRUM Consortium, UK

## Abstract

**Introduction:**

In the last 5 years, there has been a dramatic shift in the types of nicotine products being purchased. This study aimed to estimate how much users spend on types of cigarettes and alternative nicotine products (e-cigarettes, nicotine replacement therapy (NRT), heated tobacco, and nicotine pouches) and describe changes between 2018 and 2022.

**Aims and Methods:**

Monthly representative cross-sectional survey in England. 10 323 adults who smoked cigarettes or used alternative nicotine reported their average weekly expenditure on these products, adjusted for inflation.

**Results:**

Smokers spent £20.49 [95% CI = 20.09–20.91] on cigarettes each week (£27.66 [26.84–28.50] and £15.96 [15.49–16.28] among those who mainly smoked manufactured and hand-rolled cigarettes, respectively), e-cigarette users spent £6.30 [5.99–6.55] (£8.41 [7.17–9.78], £6.42 [5.58–7.39], and £5.93 [5.64–6.30] among those who mainly used disposable, pod, and refillable devices, respectively), NRT users £6.11 [5.53–6.69], and heated tobacco users £13.87 [9.58–20.09]. Expenditure on cigarettes grew by 10% from September 2018 to July 2020, then fell by 10% from July 2020 to June 2022. These changes coincided with a 13% reduction in cigarette consumption and a 14% increase in the proportion mainly smoking hand-rolled cigarettes. Expenditure on e-cigarettes was stable between 2018 and late 2020, then rose by 31% up to mid-2022. Expenditure on NRT increased slowly in 2018–2020 (+4%) and more quickly thereafter (+20%).

**Conclusions:**

Inflation-adjusted expenditure on cigarettes has fallen since 2020, such that the average smoker in England currently spends the same on cigarettes each week as in 2018. This has been achieved by smoking fewer cigarettes and switching to cheaper hand-rolled cigarettes. Expenditure on alternative nicotine has increased above inflation; users spent around a third more on these products in 2022 than between 2018–2020.

**Implications:**

People in England continue to spend substantially more on smoking cigarettes than using alternative nicotine products. The average smoker in England spends around £13 a week (~£670 a year) more than people using only e-cigarettes or NRT. The average expenditure on manufactured cigarettes is double that of hand-rolled cigarettes.

## Introduction

In England, maintaining high rates of tax on tobacco is a key strategy for reducing smoking prevalence.^1^ After concern for health, cost ranks among smokers’ leading motives for quitting,^2^ and increasing tobacco excise taxes and prices are effective in reducing population-level tobacco consumption and prevalence of use.^3–5^ Stopping smoking is difficult because of the addictive nature of nicotine delivered rapidly by cigarettes,^6^ but there are less harmful alternative nicotine products available that increase smokers’ chances of successfully quitting tobacco smoking,^7,8^ potentially saving them money.^9^ These include licensed nicotine replacement therapy (NRT) products, and e-cigarettes which are generally sold as consumer products but are recommended for cessation in the United Kingdom under NICE guidance.^10^ The alternative nicotine product market is rapidly evolving, and there have been notable shifts in the types of products and e-cigarette devices being used over recent years.^11–13^ Maintaining a price differential between smoking cigarettes and using alternative nicotine products is likely to be important in encouraging smokers to switch to less harmful products.^14–17^ Understanding how the financial impact of smoking cigarettes compares with alternative nicotine products, and the extent to which this is changing in a fast-evolving market, is therefore important for informing policy decisions and may provide useful information that could be communicated to smokers to inspire them to switch.

In 2018, we used data from the Smoking Toolkit Study, a representative survey of adults in England, to analyze comparative expenditure on cigarettes, e-cigarettes, and licensed NRT products.^9^ We found smokers reported spending on average £23.40 on smoking each week, e-cigarette users £7.60, and NRT users £8.15. Cigarette expenditure was higher among heavier smokers and lower among hand-rolled cigarette smokers and those from routine and manual occupations. Ex-smokers who had switched to e-cigarettes or NRT reported spending on average £8.03 or £10.05 on respective products each week. These data suggested the average smoker could save around two-thirds of what they were spending on smoking (~£13–15 each week) by switching to one of these less harmful nicotine products, although this will differ according to individual usage patterns.

Since this analysis was published, there have been several developments that warrant an updated examination of what people are spending on cigarettes and alternative nicotine. First, the types of devices vapers are using have changed. In 2020, there was an increase in use of pod e-cigarettes (eg, JUUL), which became more popular than modified or modular (“mod”) devices.^11^ Between 2021 and 2022, use of new disposable e-cigarettes (eg, Puff bar, Elf bar) grew rapidly, particularly among young people.^13,18^ While these new disposables are relatively cheap, retailing at around £5-7 (US$7–9), it is unclear how the average expenditure by users of these products compares with those who use other e-cigarette devices which have a higher initial cost (~£15 for a starter kit) but use low-cost e-liquid refills (~£2.95 for a 10 mL bottle).

Secondly, there are other nicotine products on the market for which we currently have no published expenditure data. One example is heated tobacco products (eg, IQOS), which are intended to heat tobacco without burning it to a high enough temperature to release aerosol.^19^ We had intended to look at expenditure on heated tobacco in 2018, but there were too few users in the three waves of data available. Recent estimates suggest heated tobacco product use remains rare,^11^ but with three and a half years’ worth of additional data now available in the Smoking Toolkit Study, we have a larger sample for analysis. Another newer product category is nicotine pouches (eg, Zyn, Nordic Spirit, and Velo), which are placed between the lip and gum where they rapidly and effectively deliver nicotine.^20^ Nicotine pouches were first introduced to European markets outside Scandinavia in 2019 and are now sold by all major tobacco companies.^21^ While prevalence of use is still relatively low (around 1 in 400 adults in Great Britain reported using them in 2020–2021), it has increased over time.^12^

Thirdly, the economic climate has been affected by Brexit, the coronavirus disease-19 pandemic, and the war in Ukraine. This has resulted in a cost-of-living crisis, with high rates of inflation and pay increases failing to keep up with rising prices.^22,23^ Given people now have less disposable income, patterns of expenditure on cigarettes and alternative nicotine products might have changed, with users either switching to lower-cost products or reducing their consumption to cut down the amount they are spending.

This study, therefore, aimed to provide up-to-date estimates of self-reported expenditure on cigarettes, e-cigarettes, and other nicotine products. Specifically, we aimed to estimate:

1. How much past-year smokers in England who use these products spend each week on average on:
  a. Cigarettes (overall and by main type of cigarettes smoked [hand-rolled, manufactured]);
  b. E-cigarettes (overall and by device type used [disposable, refillable, pod]);
  c. Licensed NRT products;
  d. Heated tobacco products;
  e. Nicotine pouches.
2. Changes in average weekly expenditure on these products changed between 2018 and 2022, before and after adjustment for inflation.
3. Differences in expenditure on these products by sociodemographic and product use characteristics.

## Method

### Preregistration

The study protocol was preregistered on Open Science Framework (https://osf.io/vqa5h/). We made three amendments to our preregistered analysis plan. First, we excluded non-cigarette smokers on account of small numbers and wide variation in types, costs, and frequency of use of other combustibles. Second, we tested the interaction between time and exclusive versus dual use to explore whether we could detect differences in trends. Third, to contextualize changes in expenditure on cigarettes and e-cigarettes, we modeled changes in cigarette consumption and prevalence of use of different types of cigarettes (manufactured, hand-rolled) among smokers and changes in prevalence of use of different types of e-cigarettes (disposable, refillable, and pod) among e-cigarette users.

### Design

Data were drawn from the Smoking Toolkit Study, a monthly cross-sectional survey of a representative sample of adults in England. The sampling methods are described in full elsewhere.^24^ Briefly, England is split into output areas, which are stratified by region and demographic characteristics before being randomly selected for inclusion on the interview list. Interviews are conducted in these selected areas until quotas based on working status, age, and gender are met. Comparisons with other national surveys and sales data indicate key variables such as sociodemographic characteristics, smoking prevalence, and cigarette consumption are nationally representative.^24,25^

Data were collected monthly through face-to-face computer-assisted interviews up to February 2020. However, social distancing restrictions under the coronavirus disease-19 pandemic meant no data were collected in March 2020, data from April 2020 onwards were collected via telephone, and the lower age bound for participation was increased from 16 to 18 years due to changes in consenting procedures. The telephone-based data collection relied upon the same combination of random location and quota sampling, and weighting approach as the face-to-face interviews and the two data collection modalities show good comparability.^26–28^

### Participants

This analysis included participants who responded to the survey between September 2018 (the first wave to ask about spending on alternative nicotine products) and June 2022 (the most recent data available at the time of analysis). We used data from past-year smokers who reported (1) current cigarette smoking and/or current use of (2) e-cigarettes, (3) any licensed NRT product, (4) heated tobacco products, or (5) nicotine pouches. We excluded participants with missing expenditure data. Participants who had not smoked in the past year were not asked the questions on expenditure so they were not eligible for inclusion. Because the sample was restricted to people aged ≥18 years when data collection switched from face-to-face to telephone interviews, we also excluded any participants aged 16–17 recruited before April 2020 for consistency (note that our previous paper^9^ included all participants ≥16 years).

### Measures

Full details of all the measures we used are available in Supplementary File 1.

#### Expenditure on Cigarettes and Alternative Nicotine Products

Weekly expenditure on cigarettes was assessed in current smokers with the question: “On average about how much per week do you think you spend on cigarettes or tobacco?.” Weekly expenditure on other nicotine products was assessed in current users with the question: “On average about how much per week do you think you spend on using this nicotine replacement product or products?.” This question followed the assessment of current use of e-cigarettes, NRT, heated tobacco products, and nicotine pouches and referred to the product(s) the participant reported using. Participants were asked to only answer questions on expenditure if they were fairly confident that they knew. Responses to both items were given to the nearest pound. Because the item on expenditure on alternative nicotine products did not differentiate between product groups, we analyzed expenditure overall (ie, average expenditure across all alternative nicotine users) and among exclusive users of e-cigarettes, NRT, heated tobacco products, and nicotine pouches. We log-transformed expenditure variables for analysis to normalize skewed distributions and reported results as geometric means (note that our previous paper^9^ reported arithmetic means, so the figures are not directly comparable). Inflation adjustment was calculated using monthly inflation figures (ie, we assumed £1 in June 2022 was equivalent to £0.99 in May 2022, £0.98 in April 2022, £0.95 in March 2022, etc.).^29^

#### Sociodemographic and Product Use Characteristics

We recorded the following:

- Among all smokers and alternative nicotine users: age, gender, occupational social grade, daily versus non-daily use, exclusive (ie, cigarettes or alternative nicotine only) versus dual use (ie, both cigarettes and alternative nicotine);
- Among smokers: Daily cigarette consumption, main type of cigarettes smoked (manufactured or hand-rolled);
- Among e-cigarette users: Main type of e-cigarettes used (disposable, refillable, or pod).

### Statistical Analysis

Analyses were done using R v.4.2.1. The Smoking Toolkit Study uses survey weights to adjust data so that the sample matches the demographic profile of England on age, social grade, region, housing tenure, ethnicity, and working status within sex.^24^

Descriptive statistics on sociodemographic and product use characteristics were calculated for each group of product users, as relevant.

Where there were sufficient data, we estimated the average (geometric mean) weekly expenditure on (1) cigarettes, (2) any alternative nicotine product, (3) e-cigarettes, (4) NRT, (5) heated tobacco, and (6) nicotine pouches by all, exclusive, and dual users of these products. This analysis was done with and without adjustment for inflation. Within each product category and user group, we ran a linear regression model with log expenditure as the outcome and time modeled using restricted cubic splines with three knots placed at the earliest, middle, and latest months. This allowed for flexible and non-linear changes in expenditure over time, while avoiding categorization.^30^ For the overall analyses, models only included time as a predictor. For analyses looking at specific subtypes of cigarettes (hand-rolled or manufactured) and e-cigarettes (disposable, refillable, or pod), models included time, sub-type, and their interaction as predictors—thus allowing time trends to differ across subtypes. Similarly, for analyses looking at differences between exclusive users and dual users, models included time, use type, and their interaction as predictors. We used predicted estimates from these models to (1) plot average weekly expenditure over the study period and (2) obtain specific predictions for current levels of expenditure (in June 2022), incorporating information from all survey months and thus increasing statistical precision. We also reported predicted expenditure for the first month of the time series (September 2018) and raw descriptive data on expenditure on each product category aggregated across participants in all survey months.

For comparison with data on expenditure on cigarettes, we estimated the average (geometric mean) daily cigarette consumption by smokers, and modeled changes over time and interactions with main type of cigarettes smoked (hand-rolled, manufactured) and use type (exclusive, dual) using linear regression with log-cigarette consumption as the outcome and survey month modeled using restricted cubic splines, as described above. We also estimated the percentage of smokers and e-cigarette users who predominantly used each product subtype (ie, hand-rolled vs. manufactured cigarettes, and disposable vs. refillable vs. pod e-cigarettes), and modeled changes over time using log-binomial regression with the use of each product subtype as the outcome and survey month modeled using restricted cubic splines.

We then used multiple linear regression models to examine independent associations between sociodemographic and product use characteristics and expenditure on (1) cigarettes among current smokers, (2) all alternative nicotine products, (3) e-cigarettes, and (4) NRT among users of these products. We had intended to examine associations with heated tobacco products and nicotine pouches, but too few participants reported using them. All models included age, gender, social grade, daily versus non-daily use, dual versus exclusive use, and survey month. The model for smokers also included cigarette type. The model for e-cigarette users also included e-cigarette device type.

## Results

There were 74 091 respondents to the survey between September 2018 and June 2022, 11 396 of whom reported current cigarette smoking or current use of an alternative nicotine product. We excluded 1073 participants (9.4%) who responded “don’t know” to the question(s) on expenditure (742 exclusive smokers, 188 exclusive users of alternative nicotine, and 143 dual users), resulting in a final sample for analysis of 10 323 respondents.

Weighted sociodemographic and product use characteristics of smokers (*n* = 9655), all alternative nicotine users (*n* = 2622), e-cigarette users (*n* = 1669), NRT users (*n* = 604), and heated tobacco users (*n* = 33) who provided expenditure data are summarized in Supplementary File 2. There were 6962 exclusive smokers, 536 exclusive users of alternative nicotine products (all of whom were ex-smokers) and 2825 dual users. Of the dual users, 739 only reported expenditure on cigarettes and 132 only reported expenditure on alternative nicotine, so were not included in analyses of expenditure on the other product. There were 47 nicotine pouch users in the sample, of whom 22 reported expenditure on alternative nicotine (the remainder were included in the sample because they were dual users who reported expenditure on cigarettes). However, all 22 used another alternative nicotine product (17 e-cigarettes, 13 NRT, and 5 heated tobacco products) as well as nicotine pouches, meaning we were unable to analyze expenditure on nicotine pouches separately.

### Overall Estimates of Expenditure

Table 1 and Supplementary File 3 (Supplementary Figure S3.1) show descriptive data (means and 95% confidence intervals) on inflation-adjusted expenditure on each product category among all users across the study period (September 2018 through June 2022, combined). Supplementary File 4 (Supplementary Table S4.1 and Figure S4.1) provides corresponding data without adjustment for inflation. Supplementary File 5 (Supplementary Table S5.1) provides data separately for exclusive users and dual users.

**Table 1.** Weekly Inflation-Adjusted Expenditure (in £) on Cigarettes and Alternative Nicotine Products: Raw Data Aggregated Across the Study Period (September 2018–June 2022) and Modeled Estimates for the First and Last Months in the Time Series

		Raw data^2^ (September 2018 – June 2022)			Modeled estimates						
					September 2018^3^			June 2022^3^			
	***N*** ^1^	**Mean** ^4^	**Lower CI**	**Upper CI**	**Mean** ^4^	**Lower CI**	**Upper CI**	**Mean** ^4^	**Lower CI**	**Upper CI**	**% change** ^5^
Cigarettes	*9655*	20.49	20.09	20.91	19.49	18.56	20.46	19.24	18.19	20.36	−1.3
Hand-rolled cigarettes	*4652*	15.96	15.49	16.28	14.35	13.52	15.22	15.03	14.03	16.11	+4.7
Manufactured cigarettes	*4561*	27.66	26.84	28.50	26.21	24.42	28.12	27.03	24.79	29.49	+3.1
All alternative nicotine products	*2622*	6.42	6.11	6.69	6.00	5.35	6.72	8.09	7.26	9.03	+34.8
E-cigarettes	*1669*	6.30	5.99	6.55	6.00	5.25	6.87	7.90	6.95	8.97	+31.7
Disposable e-cigarettes	*155*	8.41	7.17	9.78	5.31	2.67	10.57	9.65	7.46	12.50	+81.7
Refillable e-cigarettes	*1169*	5.93	5.64	6.30	5.95	5.10	6.94	7.02	5.96	8.26	+18.0
Pod e-cigarettes	*267*	6.42	5.58	7.39	5.41	3.91	7.48	7.81	5.39	11.31	+44.4
NRT	*604*	6.11	5.53	6.69	5.69	4.51	7.19	7.07	5.42	9.23	+24.3
Heated tobacco products^6^	*33*	13.87	9.58	20.09	—	—	—	—	—	—	—

CI, 95% confidence interval.

^1^Unweighted sample size.

^2^Raw weighted estimates aggregated across participants in all survey waves (September 2018 through June 2022).

^3^Data for September 2018 and June 2022 are weighted estimates from linear regression with survey month modeled non-linearly using restricted cubic splines (three knots).

^4^Geometric means are reported to account for the skewed distributions (see Supplementary File 3, Figure S3.1).

^5^Percentage change between September 2018 and June 2022.

^6^Changes in expenditure on heated tobacco products over time were not analyzed due to insufficient sample size.

Corresponding estimates of expenditure without inflation adjustment are shown in Supplementary File 4, Table S4.1.

Corresponding estimates of expenditure for exclusive users and dual users (separately) are shown in *Supplementary File* 5, *Table S5*.1 (inflation-adjusted) and *Supplementary File* 5, *Table S5*.2 (without inflation adjustment).

After adjustment for inflation, smokers spent on average £20.49 each week on cigarettes (Supplementary Figure S3.1A). Those who mainly smoked manufactured cigarettes spent around twice as much on smoking each week as those who mainly smoked hand-rolled cigarettes (£27.66 vs. £15.69; Table 1), despite smoking on average one fewer cigarette per day (geometric mean 6.62 vs. 7.69; Supplementary File 6, Table S6.1). Expenditure on cigarettes was similar among exclusive smokers and those who also used alternative nicotine (£20.29 vs. £21.12, respectively; Supplementary Table S5.1).

Alternative nicotine users spent on average £6.42 each week on alternative nicotine products (Supplementary Figure S3.1B). Exclusive users of alternative nicotine spent on average £7.69 and those who also smoked spent £6.11(Supplementary Table S5.1). Expenditure on the two most commonly used alternative nicotine products was similar: £6.30 among e-cigarette users (Supplementary Figure S3.1C) and £6.11 among NRT users (Supplementary Figure S3.1D). E-cigarette users who mainly used disposable devices spent around 40% more than those who used refillable devices (£8.41 vs. £5.93). Those who mainly used pod e-cigarette devices spent on average £6.42. Heated tobacco users spent on average £13.87 each week (Supplementary Figure S3.1E), but the wide confidence interval indicates a substantial degree of imprecision in this estimate.

### Time trends in expenditure

**Figure 1.**
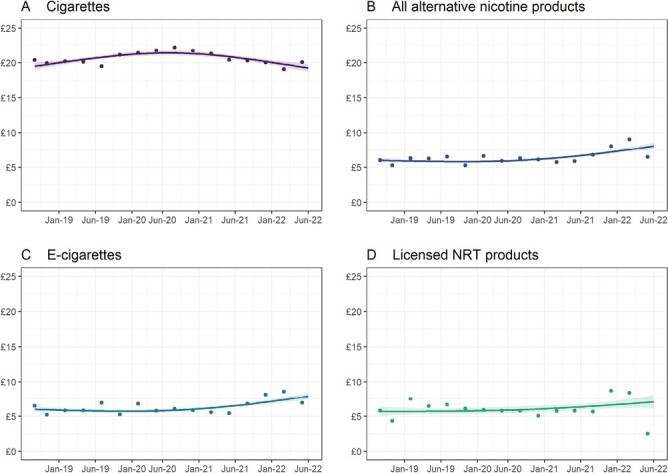
Time trends in weekly inflation-adjusted expenditure, September 2018 to June 2022. Panels show trends in weighted expenditure on (A) cigarettes by smokers and (B) all alternative nicotine products, (C) e-cigarettes, and (D) licensed nicotine replacement therapy products by users of these products. Lines represent modeled weighted geometric mean expenditure over the study period. Shaded bands represent standard errors. Points represent raw weighted (geometric mean) expenditure by quarter. Corresponding estimates of expenditure without inflation adjustment are shown in *Supplementary File* 4, *Figure S4*.2. Corresponding estimates of expenditure for exclusive users and dual users (separately) are shown in *Supplementary File* 5, *Figure S5*.1 (inflation-adjusted) and *Supplementary Figure S5*.2 (without inflation adjustment).

Figure 1 shows time trends in inflation-adjusted expenditure on each product category from September 2018 to June 2022. Table 1 summarizes modeled estimates of inflation-adjusted expenditure in the first and last months of the time series. Supplementary File 5 provides data separately for exclusive users and dual users.

**Figure 2.**
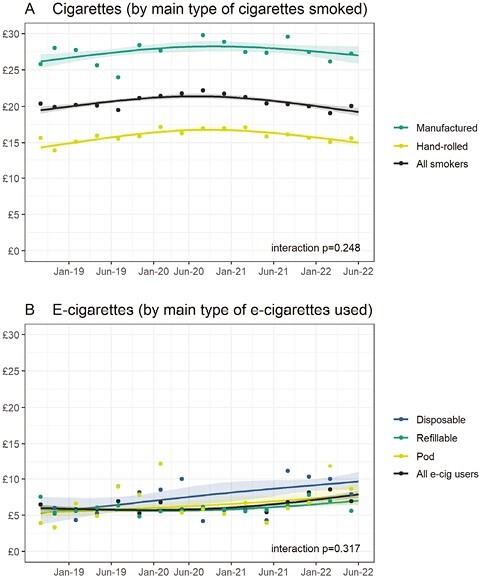
Time trends in weekly inflation-adjusted expenditure by main type of cigarettes/e-cigarettes used, September 2018 to June 2022. Panels show trends in weighted expenditure on (A) cigarettes, by all smokers and separately by main type of cigarettes smoked (hand-rolled, manufactured), and (B) e-cigarettes, by all e-cigarette users and separately by main device type used (disposable, refillable, and pod). Lines represent modeled weighted (geometric mean) expenditure over the study period. Shaded bands represent standard errors. Points represent raw weighted (geometric mean) expenditure by quarter. Corresponding estimates of expenditure without inflation adjustment are shown in *Supplementary File* 4, *Figure S4*.3.

Over the study period, geometric mean inflation-adjusted expenditure on cigarettes increased (peaking at £21.41 in July 2020) and then decreased (Figure 1A). Daily cigarette consumption was relatively stable between September 2018 and July 2020 and then decreased (Supplementary File 6). From September 2018 to June 2022, the amount the average smoker spent on smoking each week was similar (£19.49 and £19.24, respectively), despite mean daily cigarette consumption falling by 13.2% over this period (from 7.28 to 6.32 cigarettes per day; Supplementary File 6), likely reflecting prices increasing above inflation (from 38p [19.49/(7 × 7.28)] to 43p [19.24/(7 × 6.32)] per cigarette). Time trends were similar in smokers who mainly smoked hand-rolled cigarettes and those who mainly smoked manufactured cigarettes (Figure 2A; interaction *p* = .248) and within exclusive smokers and dual users (Supplementary File 5, Figure S5.1A; interaction *p* = .230). However, there was a shift in product choice over this period: the proportion of smokers who reported mainly smoking hand-rolled cigarettes increased from 48.6% in September 2018 to 55.4% in July 2022 (Supplementary File 7).

There were increases in inflation-adjusted expenditure on alternative nicotine products. Between September 2018 and June 2022, mean expenditure on all alternative nicotine products increased by 34.8% (from £6.00 to £8.09; Figure 1B). Expenditure on e-cigarettes and NRT increased by 31.7% (from £6.00 to £7.90; Figure 1C) and 24.3% (from £5.69 to £7.07; Figure 1D), respectively. Increases in expenditure on alternative nicotine products predominantly occurred in the second half of the study period (late 2020 onwards; Figure 1B-D). Time trends were similar within exclusive and dual users (Supplementary File 5, Figure S5.1B-D; interactions *p* > .230). Time trends in expenditure within e-cigarette types were comparable across devices (Figure 2B; interaction *p* = .317), although changes in the overall increase across the period may reflect increasing use of comparatively more expensive products such as disposables. There was a substantial increase in the proportion of e-cigarette users who reported mainly using disposables, from 10.2% in September 2018 to 38.5% in July 2022, which was offset by a decline in the proportion using refillable devices (from 70.1% to 56.7% over the same period), with no notable change in the proportion using pod devices (Supplementary File 7). There were too few heated tobacco users (who did not use another alternative nicotine product, *n* = 33) to analyze changes in expenditure on heated tobacco products separately.

Supplementary File 4 (Figure S4.2 and Figure S4.3) provides corresponding data on expenditure without adjustment for inflation. Trends in nominal expenditure on alternative nicotine products were similar to trends in inflation-adjusted expenditure (Supplementary Figure S4.2B-D). The trend in nominal expenditure on cigarettes increased up to late 2020 before plateauing (Supplementary Figure S4.2A).

### Associations of Expenditure With Sociodemographic and Product Use Characteristics

Table 2 shows adjusted associations of expenditure with sociodemographic and product use characteristics.

**Table 2.** Independent Associations of Weekly Inflation-Adjusted Expenditure on Cigarettes and Alternative Nicotine Products With Sociodemographic and Product Use Characteristics

	A. Cigarettes^1^		B. Alternative nicotine products^2^		C. E-cigarettes^3^		D. Licensed NRT products^4^	
	Exp. *B*^5^ (95% CI)	*p*	Exp. *B*^5^ (95% CI)	*p*	Exp. *B*^5^ (95% CI)	*p*	Exp. *B*^5^ (95% CI)	*p*
Age (per 10 years)	1.04 (1.03, 1.05)	<.001	0.90 (0.87, 0.92)	<.001	0.88 (0.85, 0.91)	<.001	0.95 (0.89, 1.00)	.073
Women (vs. men)	0.89 (0.86, 0.92)	<.001	0.84 (0.77, 0.92)	<.001	0.79 (0.72, 0.88)	<.001	0.90 (0.74, 1.09)	.268
Social grade C2DE (vs. ABC1)	1.09 (1.05, 1.12)	<.001	0.99 (0.91, 1.08)	.838	0.98 (0.89, 1.08)	.687	0.93 (0.77, 1.12)	.456
Non-daily user (vs. daily)	0.32 (0.31, 0.34)	<.001	0.71 (0.65, 0.78)	<.001	0.73 (0.65, 0.83)	<.001	0.62 (0.51, 0.75)	<.001
Dual user of cigarettes and alternative nicotine (vs. exclusive user)	1.04 (1.01, 1.08)	.026	0.88 (0.79, 0.98)	.017	0.91 (0.80, 1.03)	.122	0.87 (0.67, 1.13)	.289
Mainly smokes hand-rolled cigarettes (vs. manufactured)	0.53 (0.51, 0.55)	<.001	—	—	—	—	—	—
Main type of e-cigarette used (ref disposable)								
Refillable	—	—	—	—	0.77 (0.64, 0.92)	0.004	—	—
Pod	—	—	—	—	0.88 (0.71, 1.10)	0.259	—	—

CI, confidence interval. NRT, nicotine replacement product.

Results for each product category are from a multivariable model including all variables as listed plus survey month (modeled non-linearly using restricted cubic splines [three knots]).

^1^Among smokers.

^2^Among alternative nicotine users, including participants who reported using more than one form of alternative nicotine product.

^3^Among e-cigarette users who did not report using any other alternative nicotine product.

^4^Among NRT users who did not report using any other alternative nicotine product.

^5^Expenditure was log-transformed for analysis. Results are reported as exponentiated coefficients, which can be interpreted as geometric mean ratios. For example, the geometric mean ratio for expenditure on cigarettes among women vs. men is 0.89, which indicates women spend on average 11% less on smoking each week than men.

Men spent an average of 11% more on cigarettes than women (Table 2A). Expenditure on cigarettes was also 9% higher in smokers from social grades C2DE than ABC1, and increased by 4% for every 10 years of age. Daily smokers spent 3 times as much on cigarettes as non-daily smokers. As was observed in the unadjusted analyses, those who mainly smoked manufactured cigarettes spent almost double the amount of those smoking hand-rolled cigarettes, indicating this difference was not due to different user profiles. Expenditure on cigarettes was similar among those who exclusively smoked and those who also used alternative nicotine products.

On average, men who used alternative nicotine spent 16% more on these products than women (21% for e-cigarettes, 10% for NRT; Table 2B-D). Expenditure on alternative nicotine products was similar across social grades, and it decreased by 10% for every 10 years of age (12% for e-cigarettes, 5% for NRT). Daily users spent 29% more on alternative nicotine products than non-daily users (27% for e-cigarettes, 48% for NRT). Exclusive users of alternative nicotine product users spent 12% more on alternative nicotine products than dual users who also smoked.

E-cigarette users who mainly used disposable devices spent 23% more on e-cigarettes than those who mainly used refillable devices.

## Discussion

In England, between September 2018 and June 2022, smokers reported spending on average £20.49 on cigarettes each week after adjustment for inflation (£27.66 and £15.96 among those who mainly smoked manufactured and hand-rolled cigarettes, respectively), e-cigarette users reported spending £6.30 (£8.41, £6.42, and £5.93 among those who mainly used disposable, pod, and refillable devices, respectively), NRT users reported spending £6.11, and heated tobacco users reported spending £13.87. There was not enough data to estimate expenditure on nicotine pouches. People who used to smoke and now use e-cigarettes reported spending on average £7.32 on e-cigarettes each week, while those who now use NRT reported spending £7.24 each week. Compared with the average expenditure on smoking by those who did not use any alternative nicotine product, people using only e-cigarettes were spending approximately £12.97 a week less (£674 a year) and people using only NRT were spending approximately £13.05 a week less (£679 a year). People using only NRT and e-cigarettes would also be benefitting from using substantially less harmful products.^18^

Inflation-adjusted expenditure on cigarettes increased by 10% between September 2018 and July 2020, then decreased by 10% between July 2020 and June 2022. This meant nominal expenditure on smoking was stable in the latter period, at around £18.60 a week. Two factors that likely contributed to this are changes in the amount and types of cigarettes people smoked. The average number of cigarettes smoked per day fell over the study period, from 7.28 to 6.32, and the proportion of smokers who mainly used hand-rolled cigarettes increased, from 48.6% to 55.4%. These changes were predominantly in the latter half of the study period, coinciding with the plateau in nominal expenditure on cigarettes. This suggests there may have been a ceiling effect on expenditure, with smokers either unable or unwilling to spend any more on cigarettes each week (the current cost-of-living crisis may have been a contributing factor). To avoid increasing the amount they were spending on cigarettes as prices rose, smokers changed their behavior: smoking fewer cigarettes and switching from more expensive manufactured cigarettes to cheaper hand-rolled cigarettes. These cost-saving strategies are commonly used by smokers to mitigate the impact of price rises (eg, caused by increases in tobacco taxation or high rates of inflation) without quitting.^31–33^ Other strategies we did not assess, but which may have contributed to the observed decline in expenditure on cigarettes, include switching to a cheaper brand within the same product category, buying illicit, cross-border, or duty-free cigarettes,^31,32^ or people adjusting the way that they smoke to get the amount of nicotine they desire from fewer cigarettes.^34^ As a result of Brexit, people traveling from the United Kingdom to countries within the European Union have been able to buy and bring back duty-free cigarettes since January 2021.^35^ This change broadly coincided with the decline in cigarette expenditure we observed, suggesting Brexit may have contributed to keeping the cost of smoking stable over time—making it more affordable for people to continue to smoke and potentially undermining tobacco control policy.

Inflation-adjusted expenditure on e-cigarettes was stable between September 2018 and late 2020, then increased by a third (31%) up to June 2022. While time trends in expenditure were similar by the main type of e-cigarettes used, there was a shift in product choice in the latter part of the study period: the proportion of e-cigarette users who reported mainly using disposable devices increased substantially, the proportion using refillable devices declined, and the proportion using pod devices remained similar. This likely contributed to the increase in expenditure on e-cigarettes, given the higher average cost of disposable e-cigarettes (£5–7) compared with e-liquid refills (~£2.95 for a 10 mL bottle). Inflation-adjusted expenditure on NRT increased slowly in the first half of the study period (+4% between September 2018 and July 2020) and more quickly in the second half (+20% between July 2020 and June 2022). There were too few users in our sample to estimate time trends in expenditure on heated tobacco. The increases in expenditure on e-cigarettes and NRT suggest that any ceiling effect on expenditure on cigarettes was not experienced by alternative nicotine users, possibly because their baseline level of expenditure was lower.

Expenditure also differed by sociodemographic characteristics. Across the study period, expenditure on all products was higher among men. Expenditure on cigarettes was also higher among older smokers and those from social grades C2DE. These results are consistent with previous studies showing higher average levels of consumption in these groups (eg, ^24^). Expenditure on e-cigarettes and NRT was also higher among younger users and those who had completely stopped smoking cigarettes. It is possible that age differences are attributable to variations in product use characteristics, such as brands used, source of purchase, or frequency of use. The higher levels of expenditure on alternative nicotine products among ex-smokers than dual users may be attributable to higher consumption: these products were their only source of nicotine, while dual users also obtained nicotine from cigarettes.

It would be possible to compare expenditures on different product categories reported in the current study and estimate potential savings that smokers could make by switching to different products. Our results suggest that everything else being equal, the average smoker may be able to save a substantial amount of money if they switched to e-cigarette use at a level commensurate with the average e-cigarette user. However, differences in expenditure could be driven by underlying differences in purchasing and consumption behavior among people within the product categories. Future research should assess people who switch longitudinally and estimate average savings within users over time.

Key strengths of this study include the large, representative sample and repeat cross-sectional design. There were also limitations. Only past-year smokers were asked questions on expenditure so our analysis necessarily focused on this group. It is possible that alternative nicotine users who had not smoked in the past year may have different expenditure patterns than the groups included in this analysis. The question assessing expenditure on alternative nicotine did not distinguish between the specific products. This meant we were unable to determine the amount spent on specific products among the 12.5% of alternative nicotine users who reported using more than one alternative nicotine product. In addition, few people use heated tobacco and nicotine pouches in England^11,12^ and those who do tend to also use other nicotine products. Therefore, sample sizes for these products were small (33 and 0, respectively), meaning our data can only provide an imprecise estimate of expenditure on heated tobacco and offer no insight into nicotine pouches. As the popularity of these products in England increases, it may be possible to revisit this analysis to obtain more accurate estimates of their cost to users.

In conclusion, inflation-adjusted expenditure on cigarettes has fallen since late 2020, such that the average smoker in England currently spends the same amount on cigarettes each week as they did in 2018. Expenditure on alternative nicotine products has increased above inflation, with users spending around a third more on these products in 2022 than they did between 2018 and 2020. These changes have been driven, at least in part, by changes in consumption and product choice, with smokers reducing the number of cigarettes they smoke and switching to cheaper hand-rolled cigarettes and e-cigarette users increasingly using more expensive disposable devices.

## Supplementary Material

A Contributorship Form detailing each author’s specific involvement with this content, as well as any supplementary data, are available online at https://academic.oup.com/ntr.

ntad074_suppl_Supplementary_File_S1

ntad074_suppl_Supplementary_File_S2

ntad074_suppl_Supplementary_File_S3

ntad074_suppl_Supplementary_File_S4

ntad074_suppl_Supplementary_File_S5

ntad074_suppl_Supplementary_File_S6

ntad074_suppl_Supplementary_File_S7

## Data Availability

Data and code are available from the corresponding author on reasonable request.
