# Supplementary material for: How has Expenditure on Nicotine Products Changed in a Fast-Evolving Marketplace? A Representative Population Survey in England, 2018–2022
Source: Nicotine Tob Res. 2023 May 25;25(9):1585–93. doi: 10.1093/ntr/ntad074 (PMC10439490; doi:10.1093/ntr/ntad074)
Supplement: ntad074_suppl_Supplementary_File_S1 [file ntad074_suppl_supplementary_file_s1.docx]

# How has expenditure on nicotine products changed in a fast-evolving marketplace? A representative population survey in England, 2018-2022

Supplementary File 1: Description of measures

***Smoking status*** Participants were asked which of the following best applied to them:

1. ‘I smoke cigarettes (including hand-rolled) every day’
2. ‘I smoke cigarettes (including hand-rolled), but not every day’
3. ‘I do not smoke cigarettes at all, but I do smoke tobacco of some kind (e.g. pipe, cigar or shisha)’
4. ‘I have stopped smoking completely in the last year’
5. ‘I stopped smoking completely more than a year ago’
6. ‘I have never been a smoker (i.e. smoked for a year or more)’

Those who reported currently smoking cigarettes (responses *a*–*b*) were considered smokers (response *a* daily smokers, response *b* non-daily smokers). Those who reported stopping smoking within the last year (response *d*) were considered ex-smokers. All others (responses e–*f*) were excluded from the sample as they were not asked the questions on expenditure.

***Number and type of cigarettes smoked*** Smokers were asked to report the number of cigarettes they usually smoke each day or each week (as preferred) and how many of these they think are hand-rolled. We log-transformed daily cigarette consumption for analysis to normalise the skewed distribution and reported results as geometric means for ease of interpretation. We defined hand-rolled users as those reporting at least 50% of their total cigarette consumption is hand-rolled. This definition has been used in previous studies (1,2) and allows inclusion of non-daily smokers and those who smoke both hand-rolled and manufactured cigarettes. In addition, it is similar to the definition of type of e-cigarettes used, which asks about the type participants ‘mainly’ use (see below).

***Use of alternative nicotine products*** Participants were asked a series of questions about whether they currently use e-cigarettes, licensed NRT products, heated tobacco products, and nicotine pouches to cut down the amount they smoke, in situations when they are not allowed to smoke, to help them stop smoking, or for any other reason at all. Responses were categorised as follows:

- *E-cigarette user* – ‘electronic cigarette’ or ‘JUUL’
- *Licensed NRT product user* – ‘nicotine gum’, ‘nicotine lozenge’, ‘nicotine patch’, ‘nicotine inhaler/inhalator’, ‘nicotine mouth spray’
- *Heated tobacco product user* – ‘heat-not-burn cigarette (e.g. iQOS with HEETS, heatsticks)’
- *Nicotine pouch user* – ‘tobacco-free nicotine pouch/pod or 'white pouches' that you place on your gum’

A follow-up question asked: ‘How many times per day on average do you use your nicotine replacement product or products?’ Those who reported using their product(s) at least once a day were considered daily users and those who used them less than once a day were considered non-daily users.

***Dual use of cigarettes and alternative nicotine products*** Participants who reported both smoking and using any alternative nicotine product were considered ‘dual users’.

***Type of e-cigarettes used*** E-cigarette users were asked about the specific device(s) they use: ‘Which of the following do you mainly use…?’ They could respond:

- *Disposable* – ‘a disposable e-cigarette or vaping device (non-rechargeable)’
- *Refillable* – ‘an e-cigarette or vaping device with a tank that you refill with liquids (rechargeable)’ or ‘a modular system that you refill with liquids (you use your own combination of separate devices: batteries, atomizers, etc.)’
- *Pod* – ‘an e-cigarette or vaping device that uses replaceable pre-filled cartridges (rechargeable)’

***Expenditure on cigarettes and alternative nicotine products*** Weekly expenditure on cigarettes was assessed in current smokers with the question: ‘On average about how much per week do you think you spend on cigarettes or tobacco?’. Weekly expenditure on other nicotine products was assessed in current users with the question: ‘On average about how much per week do you think you spend on using this nicotine replacement product or products?’. This question followed the assessment of current use of e-cigarettes, NRT, heated tobacco products, and nicotine pouches and referred to the product(s) the participant reported using. Participants were asked to only answer questions on expenditure if they were fairly confident that they knew. Responses to both items were given to the nearest pound. Because the item on expenditure on alternative nicotine products did not differentiate between the product groups, we analysed expenditure overall (i.e., average expenditure across all alternative nicotine product users) and among exclusive users of e-cigarettes, NRT, heated tobacco products, and nicotine pouches. We log-transformed expenditure variables for analysis to normalise skewed distributions and reported results as geometric means for ease of interpretation (note that our previous paper (3) reported arithmetic means, so the figures are not directly comparable). Inflation adjustment was calculated using monthly inflation figures (i.e., we assumed £1 in June 2022 was equivalent to £0.99 in May 2022, £0.98 in April 2022, £0.95 in March 2022, etc.) (4).

***Sociodemographic characteristics*** Age, gender, and occupational social grade were recorded. Age was analysed as a continuous variable. Gender was self-reported as man, woman, or in another way. Those who identified in another way were included in the overall sample but excluded from analyses by gender due to low numbers. Social grade was categorised as ABC1, which includes managerial, professional, and upper supervisory occupations and C2DE, which includes manual routine, semi-routine, lower supervisory, and long-term unemployed.

**References**

1. Young D, Yong HH, Borland R, Shahab L, Hammond D, Cummings KM, et al. Trends in Roll-Your-Own Smoking: Findings from the ITC Four-Country Survey (2002–2008) [Internet]. Journal of Environmental and Public Health. 2012 [cited 2018 May 24]. Available from: https://www.hindawi.com/journals/jeph/2012/406283/

2. Jackson SE, Shahab L, Garnett C, Brown J. Trends in and correlates of use of roll-your-own cigarettes: a population study in England 2008-2017. Nicotine & Tobacco Research. 2019;

3. Jackson SE, Shahab L, Kock L, West R, Brown J. Expenditure on smoking and alternative nicotine delivery products: a population survey in England. Addiction. 2019;114(11):2026–36.

4. Hargreaves Lansdown. Inflation calculator [Internet]. Hargreaves Lansdown. [cited 2022 Jul 25]. Available from: https://www.hl.co.uk/tools/calculators/inflation-calculator
