# Supplementary material for: How has Expenditure on Nicotine Products Changed in a Fast-Evolving Marketplace? A Representative Population Survey in England, 2018–2022
Source: Nicotine Tob Res. 2023 May 25;25(9):1585–93. doi: 10.1093/ntr/ntad074 (PMC10439490; doi:10.1093/ntr/ntad074)
Supplement: ntad074_suppl_Supplementary_File_S2 [file ntad074_suppl_supplementary_file_s2.docx]

# How has expenditure on nicotine products changed in a fast-evolving marketplace? A representative population survey in England, 2018-2022

Supplementary File 2: Sample characteristics

**Table S2.1.** Sample characteristics in relation to product use

**Table S2.1.** Sample characteristics in relation to product use

|  | **Smokers** | **Alternative nicotine users^1^** | **E-cigarette users^2^** | **NRT users^2^** | **Heated tobacco product users^2^** |
| --- | --- | --- | --- | --- | --- |
| *Unweighted* *N* | *9655* | *2622* | *1669* | *604* | *33* |
|  |  |  |  |  |  |
| Age (years) | 41.9 (16.4) | 39.7 (15.5) | 37.9 (14.8) | 44.9 (16.5) | 35.7 (13.1) |
| Gender |  |  |  |  |  |
| Men | 52.8% | 56.3% | 56.7% | 51.3% | 43.5% |
| Women | 46.7% | 43.0% | 42.6% | 47.8% | 56.5% |
| In another way | 0.5% | 0.7% | 0.7% | 1.0% | 0.0% |
| Social grade C2DE | 60.9% | 56.2% | 55.7% | 54.5% | 57.5% |
| Dual user of cigarettes and alternative nicotine | 28.1% | 79.0% | 78.3% | 82.5% | 55.2% |
| Number of cigarettes smoked per day^3,4^ | 7.1 (2.8) | 6.8 (2.9) | 6.0 (3.0) | 8.2 (2.3) | 8.6 (2.1) |
| Non-daily smoker^3^ | 17.9% | 16.6% | 19.2% | 10.4% | 8.5% |
| Mainly smokes hand-rolled cigarettes^3^ | 51.2% | 36.9% | 37.3% | 37.2% | 10.8% |
| Number of alternative nicotine products used |  |  |  |  |  |
| 0 | 71.9% | - | - | - | - |
| 1 | 24.7% | 87.5% | 100.0% | 100.0% | 100.0% |
| 2 | 3.1% | 11.5% | - | - | - |
| 3/4 | 0.2% | 1.0% | - | - | - |
| E-cigarette user | 20.2% | 76.0% | 100.0% | - | - |
| Main type of e-cigarette device used^5^ |  |  |  |  |  |
| Disposable | 9.5% | 10.4% | 9.6% | - | - |
| Refillable | 72.5% | 72.7% | 73.8% | - | - |
| Pod | 18.0% | 16.9% | 16.5% | - | - |
| NRT user | 10.4% | 33.4% | - | 100.0% | - |
| Heated tobacco product user | 0.6% | 3.0% | - | - | 100.0% |
| Nicotine pouch user | 0.5% | 1.0% | - | - | - |
| Non-daily alternative nicotine user^6^ | 46.2% | 38.9% | 32.3% | 55.2% | 17.9% |
| Data are shown as weighted means (SD) or weighted percentages.  There were some missing data for some variables (4.2% cigarettes smoked per day, 4.9% mainly smokes hand-rolled cigarettes, 13.0% main type of e-cigarette device used, 16.1% non-daily alternative nicotine use). Valid percentages are shown.  NRT, nicotine replacement product.  ^1^ Includes participants who reported using more than one form of alternative nicotine product (unweighted *n*=316). ^2^ Excludes participants who reported using any other form of alternative nicotine. ^3^ Among smokers. ^4^ Geometric means are reported to account for the skewed distribution. ^5^ Among e-cigarette users. ^6^ Among alternative nicotine users. | | | | | |
