# Supplementary material for: How has Expenditure on Nicotine Products Changed in a Fast-Evolving Marketplace? A Representative Population Survey in England, 2018–2022
Source: Nicotine Tob Res. 2023 May 25;25(9):1585–93. doi: 10.1093/ntr/ntad074 (PMC10439490; doi:10.1093/ntr/ntad074)
Supplement: ntad074_suppl_Supplementary_File_S3 [file ntad074_suppl_supplementary_file_s3.docx]

# How has expenditure on nicotine products changed in a fast-evolving marketplace? A representative population survey in England, 2018-2022

Supplementary File 3: descriptive data on inflation-adjusted expenditure

**Figure S3.1.** Distributions of weekly inflation-adjusted expenditure aggregated across the study period (September 2018-June 2022)


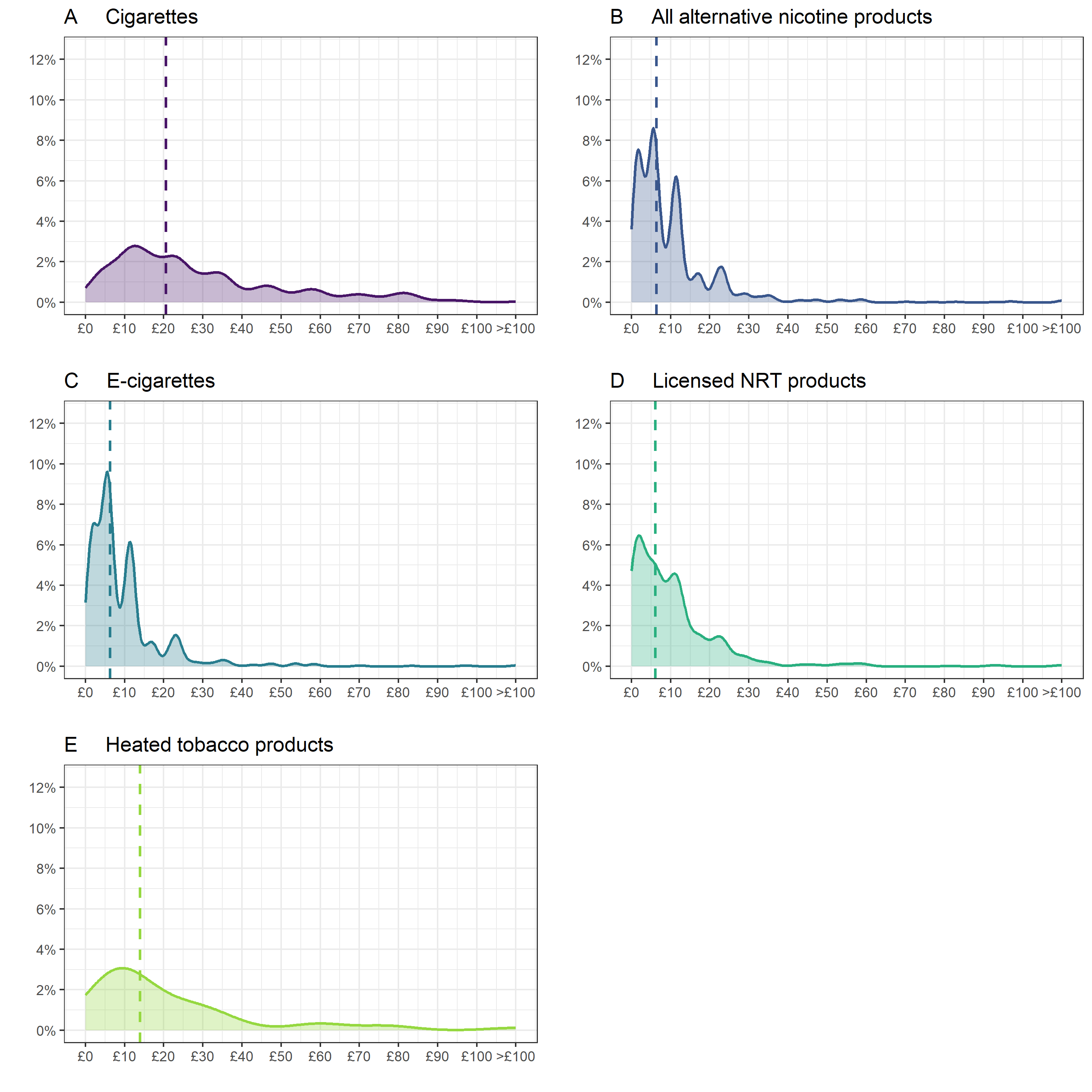


**Figure S3.1. Distributions of weekly inflation-adjusted expenditure aggregated across the study period (September 2018-June 2022).** Panels show weighted expenditure on (A) cigarettes by smokers and (B) all alternative nicotine products, (C) e-cigarettes, (D) licensed NRT products, and (E) heated tobacco products by users of these products. The dashed vertical line indicates the weighted geometric mean level of expenditure for that product category. Corresponding data on expenditure without inflation adjustment are shown in *Supplementary File 4, Figure S4.1*.
