# Supplementary material for: How has Expenditure on Nicotine Products Changed in a Fast-Evolving Marketplace? A Representative Population Survey in England, 2018–2022
Source: Nicotine Tob Res. 2023 May 25;25(9):1585–93. doi: 10.1093/ntr/ntad074 (PMC10439490; doi:10.1093/ntr/ntad074)
Supplement: ntad074_suppl_Supplementary_File_S4 [file ntad074_suppl_supplementary_file_s4.docx]

# How has expenditure on nicotine products changed in a fast-evolving marketplace? A representative population survey in England, 2018-2022

Supplementary File 4: Data on all users without adjustment for inflation

**Figure S4.1.** Distributions of nominal weekly expenditure aggregated across the study period (September 2018-June 2022)

**Table S4.1.** Nominal weekly expenditure (in £) on cigarettes and alternative nicotine products: raw data aggregated across the study period (September 2018 – June 2022) and modelled estimates for the first and last months in the time series

**Figure S4.2.** Time trends in nominal weekly expenditure, September 2018 to June 2022

**Figure S4.3.** Time trends in nominal weekly expenditure by main type of cigarettes/e-cigarettes used, September 2018 to June 2022


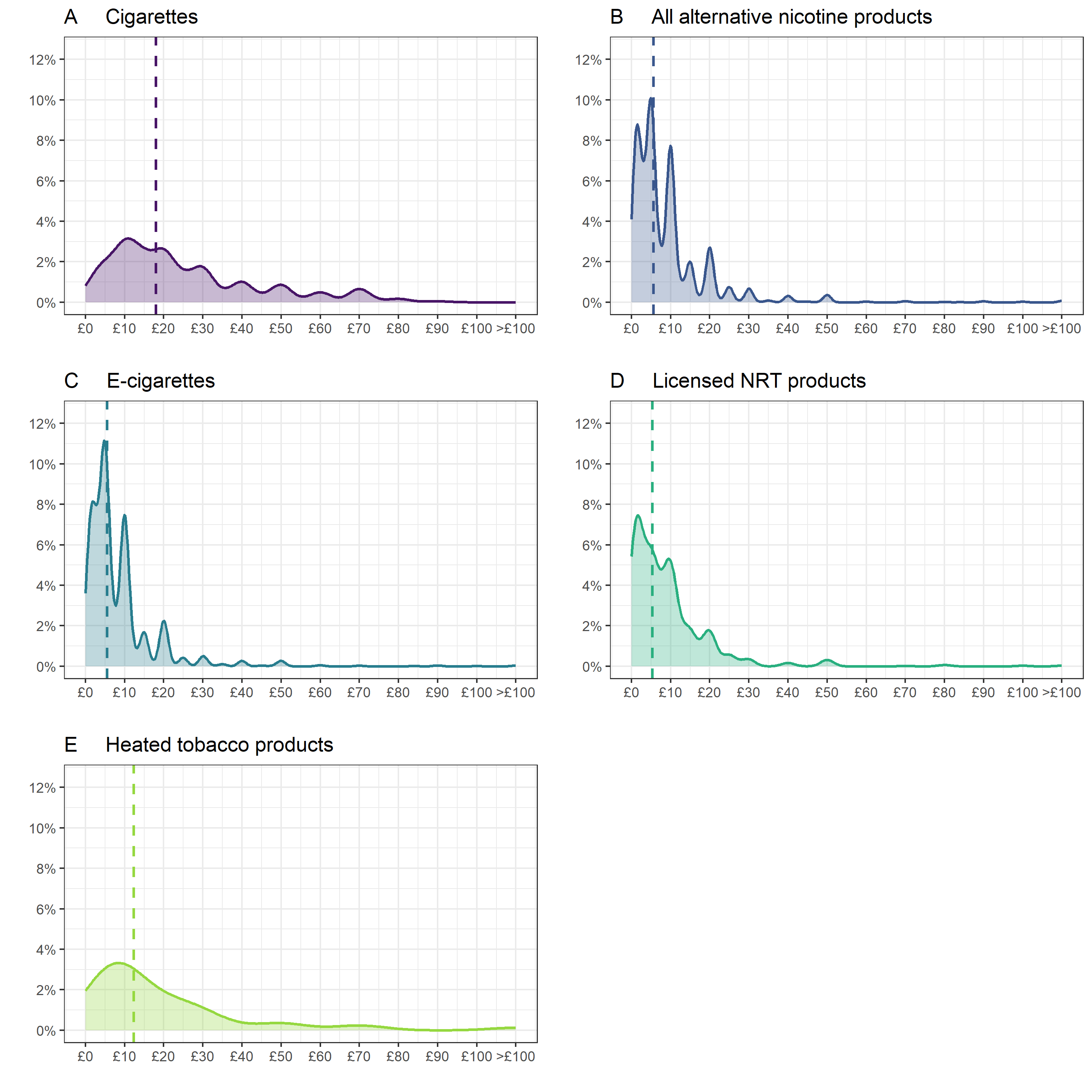


**Figure S4.1. Distributions of nominal weekly expenditure aggregated across the study period (September 2018-June 2022).** Panels show weighted expenditure on (A) cigarettes by smokers and (B) all alternative nicotine products, (C) e-cigarettes, (D) licensed NRT products, and (E) heated tobacco products by users of these products. The dashed vertical line indicates the weighted geometric mean level of expenditure for that product category.

**Table S4.1.** Nominal weekly expenditure (in £) on cigarettes and alternative nicotine products: raw data aggregated across the study period (September 2018 – June 2022) and modelled estimates for the first and last months in the time series

|  |  | **Raw data^2^**  **(September 2018** – **June 2022)** | | |  | | **Modelled estimates** | | | | | | | | | |
| --- | --- | --- | --- | --- | --- | --- | --- | --- | --- | --- | --- | --- | --- | --- | --- | --- |
|  |  |  |  |  |  | | **September 2018^3^** | | |  | | **June 2022^3^** | | |  |  |
|  | ***N*^1^** | **Mean^4^** | **Lower CI** | **Upper CI** |  | **Mean^4^** | | **Lower CI** | **Upper CI** |  | **Mean^4^** | | **Lower CI** | **Upper CI** |  | **% change^5^** |
| Cigarettes | *9655* | 17.99 | 17.64 | 18.36 |  | 16.44 | | 15.66 | 17.26 |  | 18.66 | | 17.64 | 19.74 |  | +13.5 |
| Hand-rolled cigarettes | *4652* | 13.87 | 13.60 | 14.30 |  | 12.11 | | 11.42 | 12.85 |  | 14.59 | | 13.61 | 15.63 |  | +20.5 |
| Manufactured cigarettes | *4561* | 24.05 | 23.34 | 24.78 |  | 22.10 | | 20.59 | 23.72 |  | 26.18 | | 24.00 | 28.55 |  | +18.5 |
| All alternative nicotine products | *2622* | 5.58 | 5.37 | 5.81 |  | 5.05 | | 4.51 | 5.65 |  | 7.69 | | 6.90 | 8.58 |  | +52.3 |
| E-cigarettes | *1669* | 5.47 | 5.21 | 5.75 |  | 5.05 | | 4.42 | 5.78 |  | 7.51 | | 6.61 | 8.54 |  | +48.7 |
| Disposable e-cigarettes | *155* | 7.61 | 6.49 | 8.94 |  | 4.51 | | 2.27 | 8.99 |  | 9.25 | | 7.15 | 11.97 |  | +105.1 |
| Refillable e-cigarettes | *1169* | 5.21 | 4.90 | 5.47 |  | 5.00 | | 4.29 | 5.83 |  | 6.66 | | 5.65 | 7.83 |  | +33.2 |
| Pod e-cigarettes | *267* | 5.64 | 4.90 | 6.49 |  | 4.56 | | 3.29 | 6.31 |  | 7.45 | | 5.14 | 10.79 |  | +63.4 |
| NRT | *604* | 5.31 | 4.81 | 5.87 |  | 4.78 | | 3.79 | 6.04 |  | 6.69 | | 5.13 | 8.73 |  | +40.0 |
| Heated tobacco products^6^ | *33* | 12.30 | 8.50 | 17.81 |  | - | | - | - |  | - | | - | - |  | - |

CI, 95% confidence interval.

^1^ Unweighted sample size.

^2^ Raw weighted estimates aggregated across participants in all survey waves (September 2018 through June 2022).

^3^ Data for September 2018 and June 2022 are weighted estimates from linear regression with survey month modelled non-linearly using restricted cubic splines (three knots).

^4^ Geometric means are reported to account for the skewed distributions (see *Figure S4.1*).

^5^ Percentage change between September 2018 and June 2022.

^6^ Changes in expenditure on heated tobacco products over time were not analysed due to insufficient sample size.


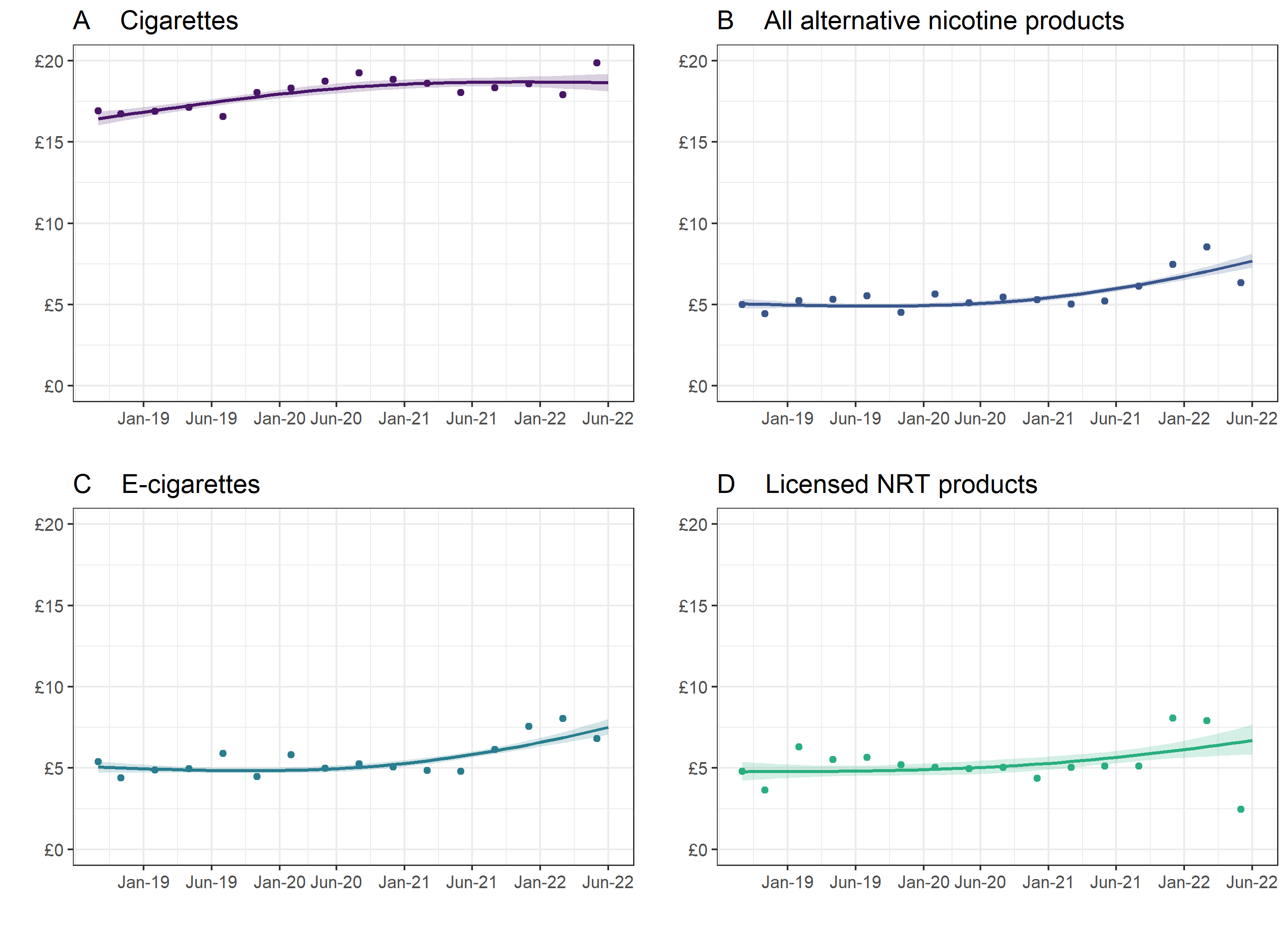


**Figure S4.2. Time trends in nominal weekly expenditure, September 2018 to June 2022**. Panels show trends in weighted expenditure on (A) cigarettes by smokers and (B) all alternative nicotine products, (C) e-cigarettes, and (D) licensed NRT products by users of these products. Lines represent modelled weighted (geometric mean) expenditure over the study period. Shaded bands represent standard errors. Points represent raw weighted (geometric mean) expenditure by quarter.

**
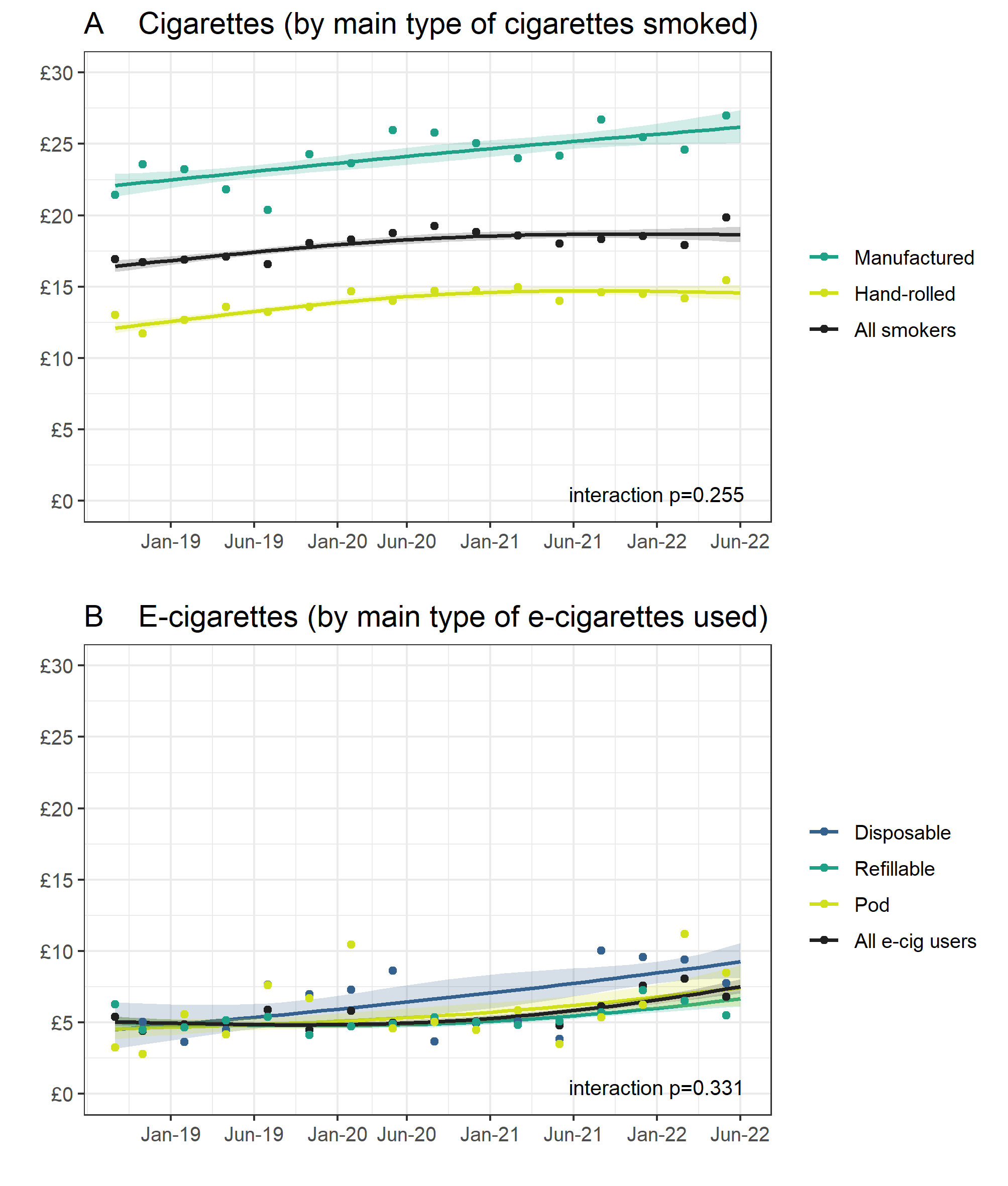
**

**Figure S4.3.** **Time trends in nominal weekly expenditure by main type of cigarettes/e-cigarettes used, September 2018 to June 2022.** Panels show trends in weighted expenditure on (A) cigarettes, by all smokers and separately by main type of cigarettes smoked (hand-rolled, manufactured), and (B) e-cigarettes, by all e-cigarette users and separately by main device type used (disposable, refillable, pod). Lines represent modelled weighted (geometric mean) expenditure over the study period. Lines represent modelled weighted (geometric mean) expenditure over the study period. Shaded bands represent standard errors. Points represent raw weighted (geometric mean) expenditure by quarter.
