# Supplementary material for: How has Expenditure on Nicotine Products Changed in a Fast-Evolving Marketplace? A Representative Population Survey in England, 2018–2022
Source: Nicotine Tob Res. 2023 May 25;25(9):1585–93. doi: 10.1093/ntr/ntad074 (PMC10439490; doi:10.1093/ntr/ntad074)
Supplement: ntad074_suppl_Supplementary_File_S6 [file ntad074_suppl_supplementary_file_s6.docx]

# How has expenditure on nicotine products changed in a fast-evolving marketplace? A representative population survey in England, 2018-2022

Supplementary File 6: Data on changes in cigarette consumption over the study period

**Figure S6.1.** Time trends in daily cigarette consumption among smokers, September 2018 to June 2022

**Table S6.1.** Nominal weekly expenditure (in £) on cigarettes and alternative nicotine products: raw data aggregated across the study period (September 2018 – June 2022) and modelled estimates for the first and last months in the time series


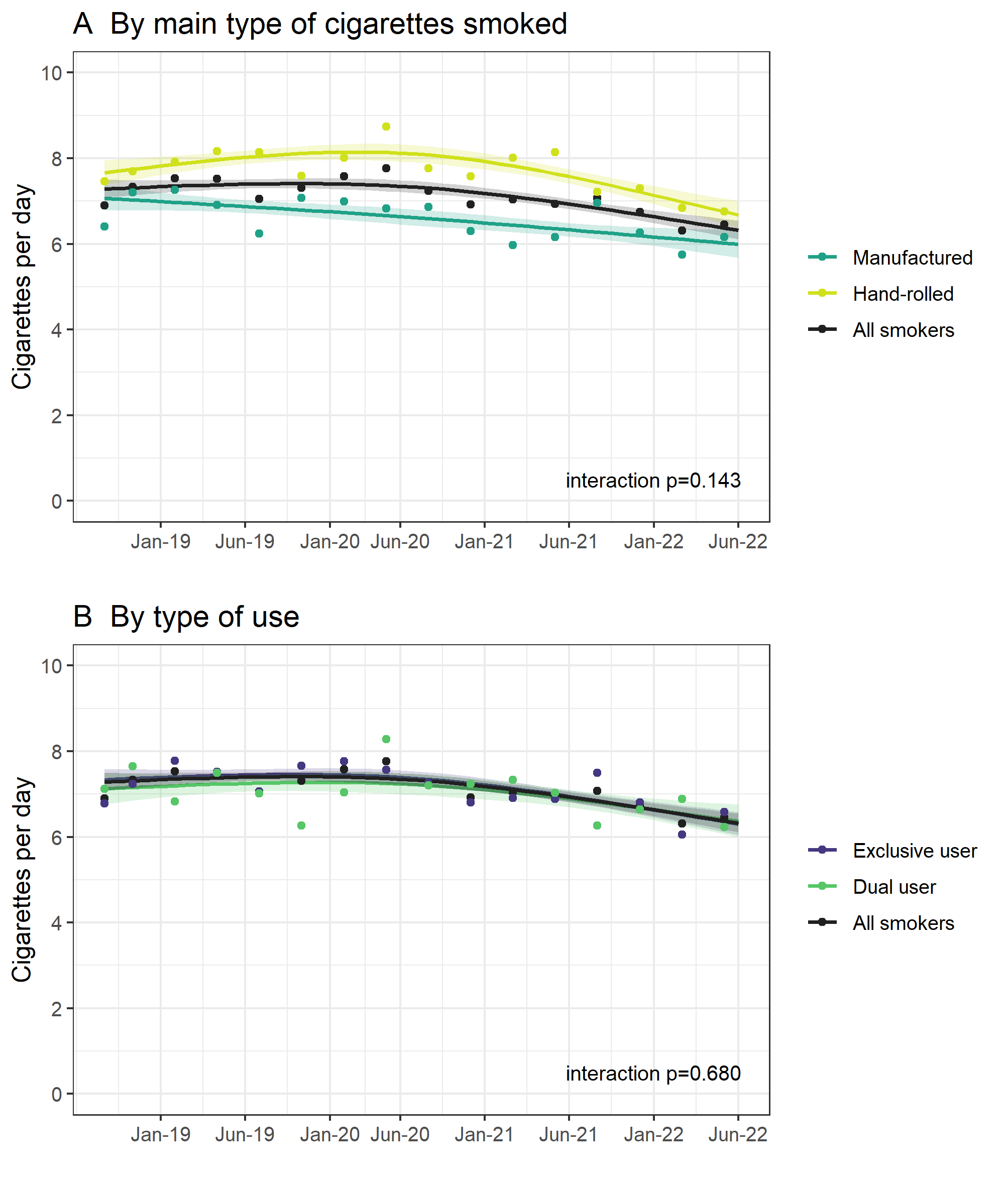


**Figure S6.1. Time trends in daily cigarette consumption among smokers, September 2018 to June 2022**. Panels show trends in daily cigarette consumption by all smokers and (A) main type of cigarettes smoked (hand-rolled, manufactured) and (B) type of use (exclusive, dual). Lines represent modelled weighted (geometric mean) daily cigarette consumption over the study period. Shaded bands represent standard errors. Points represent raw weighted (geometric mean) daily cigarette consumption by quarter.

**Table S6.1.** Daily cigarette consumption among smokers: raw data aggregated across the study period (September 2018 – June 2022) and modelled estimates for the first and last months in the time series

|  |  | **Raw data^2^**  **(September 2018** – **June 2022)** | | |  | | **Modelled estimates** | | | | | | | | | | |
| --- | --- | --- | --- | --- | --- | --- | --- | --- | --- | --- | --- | --- | --- | --- | --- | --- | --- |
|  |  |  |  |  |  | | **September 2018^3^** | | |  | | **June 2022^3^** | | |  | |  |
|  | ***N*^1^** | **Mean^4^** | **Lower CI** | **Upper CI** |  | **Mean^4^** | | **Lower CI** | **Upper CI** |  | **Mean^4^** | | **Lower CI** | **Upper CI** |  | **% change^5^** | |
| All smokers | 9427 | 7.10 | 6.96 | 7.32 |  | 7.28 | | 6.89 | 7.7 |  | 6.32 | | 5.9 | 6.77 |  | -13.2 | |
| Hand-rolled cigarette smokers | 4652 | 7.69 | 7.46 | 7.92 |  | 7.66 | | 7.12 | 8.24 |  | 6.68 | | 6.09 | 7.32 |  | -12.8 | |
| Manufactured cigarette smokers | 4561 | 6.62 | 6.36 | 6.82 |  | 7.06 | | 6.51 | 7.67 |  | 5.99 | | 5.39 | 6.66 |  | -15.2 | |

CI, 95% confidence interval.

^1^ Unweighted sample size.

^2^ Raw weighted estimates aggregated across participants in all survey waves (September 2018 through June 2022).

^3^ Data for September 2018 and June 2022 are weighted estimates from linear regression with survey month modelled non-linearly using restricted cubic splines (three knots).

^4^ Geometric means are reported to account for the skewed distribution.

^5^ Percentage change between September 2018 and June 2022.
