# Supplementary material for: How has Expenditure on Nicotine Products Changed in a Fast-Evolving Marketplace? A Representative Population Survey in England, 2018–2022
Source: Nicotine Tob Res. 2023 May 25;25(9):1585–93. doi: 10.1093/ntr/ntad074 (PMC10439490; doi:10.1093/ntr/ntad074)
Supplement: ntad074_suppl_Supplementary_File_S7 [file ntad074_suppl_supplementary_file_s7.docx]

# How has expenditure on nicotine products changed in a fast-evolving marketplace? A representative population survey in England, 2018-2022

Supplementary File 7: Data on changes in use of cigarette and e-cigarette types over the study period

**Figure S7.1.** Time trends in main types of cigarettes and e-cigarettes used, September 2018 to June 2022

**Table S7.1.** Main types of cigarettes and e-cigarettes used: raw data aggregated across the study period (September 2018 – June 2022) and modelled estimates for the first and last months in the time series


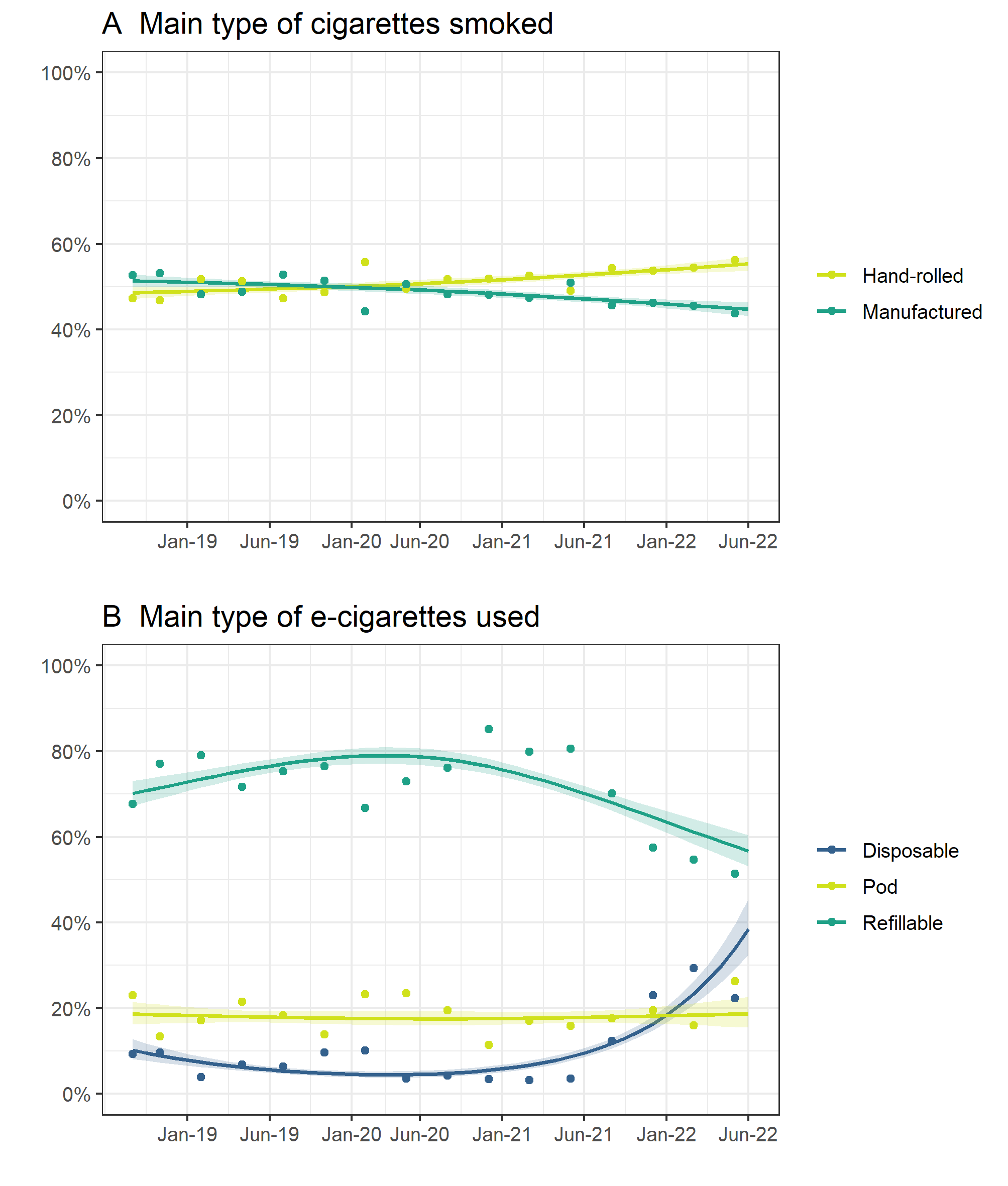


**Figure S7.1. Time trends in main types of cigarettes and e-cigarettes used, September 2018 to June 2022**. Panels show trends in the percentage of (A) smokers who reported mainly using hand-rolled vs. manufactured cigarettes and (B) e-cigarette users who reported mainly using disposable vs. refillable vs. pod devices. Lines represent modelled weighted percentages over the study period. Shaded bands represent standard errors. Points represent raw weighted percentages by quarter.

**Table S7.1.** Main types of cigarettes and e-cigarettes used: raw data aggregated across the study period (September 2018 – June 2022) and modelled estimates for the first and last months in the time series

|  |  | **Raw data^2^**  **(September 2018** – **June 2022)** | | |  | | **Modelled estimates** | | | | | | | | | | |
| --- | --- | --- | --- | --- | --- | --- | --- | --- | --- | --- | --- | --- | --- | --- | --- | --- | --- |
|  |  |  |  |  |  | | **September 2018^3^** | | |  | | **June 2022^3^** | | |  | |  |
|  | ***N*^1^** | **%^4^** | **Lower CI** | **Upper CI** |  | **%^4^** | | **Lower CI** | **Upper CI** |  | **%^4^** | | **Lower CI** | **Upper CI** |  | **% change^5^** | |
| Smokers | *9213* |  |  |  |  |  | |  |  |  |  | |  |  |  |  | |
| Hand-rolled cigarettes | - | 51.2 | 50.1 | 52.3 |  | 48.6 | | 45.9 | 51.4 |  | 55.4 | | 52.2 | 58.7 |  | +14.0 | |
| Manufactured cigarettes | - | 48.8 | 47.7 | 49.9 |  | 51.4 | | 48.6 | 54.3 |  | 44.8 | | 41.8 | 48.0 |  | -12.8 | |
| E-cigarette users | *1640* |  |  |  |  |  | |  |  |  |  | |  |  |  |  | |
| Disposable | - | 9.6 | 8.0 | 11.2 |  | 10.2 | | 6.5 | 15.9 |  | 38.5 | | 27.7 | 53.4 |  | +277.5 | |
| Refillable | - | 73.8 | 71.5 | 76.2 |  | 70.1 | | 64.6 | 76.1 |  | 56.7 | | 50.0 | 64.2 |  | -19.1 | |
| Pod | - | 16.5 | 14.6 | 18.5 |  | 18.6 | | 14.2 | 24.5 |  | 18.7 | | 12.9 | 27.1 |  | +0.5 | |

CI, 95% confidence interval.

^1^ Unweighted sample size.

^2^ Raw weighted estimates aggregated across participants in all survey waves (September 2018 through June 2022).

^3^ Data for September 2018 and June 2022 are weighted estimates from log-binomial regression with survey month modelled non-linearly using restricted cubic splines (three knots).

^4^ Geometric means are reported to account for the skewed distribution.

^5^ Percentage change between September 2018 and June 2022.
